# Mind your language: enhancing medical student learning during non-English language consultations

**DOI:** 10.3389/fmed.2025.1550101

**Published:** 2025-07-02

**Authors:** Enam Haque, Thulasi Naveenan, Genevieve Shimwell, Jasmin Farikullah, Rachel Lindley, Helen Marsden

**Affiliations:** Division of Medical Education, School of Medical Sciences, Faculty of Biology, Medicine and Health, The University of Manchester, Manchester, United Kingdom

**Keywords:** diversity, diversity and inclusion, communication skills, ethnic minority health, GP placements, undergraduate medical education

## Abstract

General Practice provides an excellent opportunity for students to see patients with undifferentiated presentations and to acknowledge how General Practitioners (GPs) deal with medical complexity, uncertainty and psycho-social issues facing patients. It is essential that students have experience of diverse patient groups, using interpreters to help with language barriers. However, many practices have GPs that speak multiple languages, and patients appreciate the opportunity to speak their own language. The challenge for students is understanding what is happening when observing these consultations. The Community Team in a UK medical school was aware of the issues, through student evaluation data. To address this, they developed a protocol to support GP Clinical Placement Supervisors (CPS) to ensure the best clinical experience for their students, particularly in practices where consultations were not commonly delivered in English. This work developed into CPS training delivered locally and nationally at other medical schools in the UK. It also led to development of an experiential learning session, where students attended a practice with non-English consultations in the morning, and then reflected on the experience in the afternoon. It now forms a core part of the student curricular content in the Year 4 GP block. This paper highlights the journey to ensure that students can have meaningful learning, in environments where language may be a perceived barrier. It has enabled our students to appreciate the diversity and rich culture of our patients and take forward the learning gleamed from the complexities of non-English consultations.

## Introduction

The United Kingdom is an increasingly diverse country to live in, with 18.3% of the England and Wales population are currently non-White, an increase from 14% in 2011 (1). 8.9% of residents are also reported to have a non-English language as their main spoken language, although only 17.1% of these are not proficient in English and only 3.1% cannot speak English at all. The main non-English languages spoken are Polish, Romanian, Punjabi, and Urdu. In Wales, the Welsh language is spoken by 17.8% of people aged three and over (2).

Despite the UK growing ever more multicultural, many ethnic minority groups in the UK face socioeconomic disadvantage, due to structural racism and other factors, leading to health inequalities (3). Al Shamsi et al. (4) conducted a systematic review looking at language barriers and found a negative impact for both patients and clinicians. This included stress and poor decision making and management for clinicians, and access barriers and dissatisfaction with healthcare for patients. Communication errors were found in a study to be a major contributor in almost 70% of adverse patient events and limited English proficiency (LEP) in another study led to more emergency readmissions, greater adverse events, and poorer patient experiences (5, 6). Fox et al. (6) found that over 50% of secondary care staff in the United States of America did not feel able to form good therapeutic relationships with patients with LEP. In a study by Pandey et al. (7), patients felt that language barriers led to them struggling to access services and to build rapport with providers, while healthcare providers were concerned that language barriers negatively impacted patients’ management of their health, leading to worse health outcomes. A patient study assessing language services provision in primary and hospital care in Manchester reported “communication difficulties” as the greatest barrier to healthcare access at multiple points along their journey from booking appointments, during consultations and when obtaining informed patient consent (8).

## Background and rationale

The General Medical Council (9) in their Good Medical Practice document have outlined “*You must take steps to meet patients’ language and communication needs, so you can support them to engage in meaningful dialog and make informed decisions about their care*.” This is addressed through use of interpreters and the NHS commissioning guide for translation services states that “*Patients should be able to access primary care services in a way that ensures their language and communication requirements do not prevent them receiving the same quality of healthcare as others*.” (10) Using interpreters has been shown to positively impact on consultations with patients who do not speak a native language, with the greatest benefit from a professional interpreter being in the same room as the clinician (11). Conversely, staff working with asylum seekers in UK primary care services noted the negative impact on patient safety of inadequate interpreter provision, when patients relied on family members, hand gestures and apps as a “workaround” (12).

Freeman et al. (13) conducted a cross-sectional survey of consultations in non-English languages between patients and clinicians and found that patients benefited from this. Patients have commented positively on clinicians speaking the same language as them, with some actively seeking professionals with these qualities (7). However, Matras and Gaiser (8) interviewed bilingual General Practitioners (GPs) and noted their reluctance to consult in their “home language.” GPs felt it changed the dynamic and expectations of the consultation, with negative outcomes for both patient and practitioner. They saw the greater value in having multilingual staff working in reception and administrative teams to help patient’s overcome barriers. Additionally, Maul et al. (14) found that there was little in the way of an objective decision-making process to help bilingual staff to decide whether to use an interpreter or rely on their own skills.

Addressing language barriers is one part of improving consultations with patients from ethnic minority backgrounds. Another is ensuring that the clinician applies cultural humility in the interaction. Tervalon and Murray-García (15) defined cultural humility as “*a lifelong commitment to self-evaluation and self-critique, to redressing the power imbalances in the patient-physician dynamic*.”

General Practice (GP) is a rich environment for undergraduate medical teaching. A systematic review noted that clinical experience in the community provided more learning opportunities, and a chance for students to explore social and cultural factors affecting a patient (16). It is becoming increasingly important that UK medical students and educators have the tools to maximize learning experiences in consultations where a non-English language is spoken. A recent focus group looking at medical student experiences of interpreter consultations, suggested the need for more cultural awareness and training for medical students to work with interpreters in medical programmes (17). McEvoy et al. (18) developed structured teaching for students on speaking with patients with limited English, and using interpreters, and noted students felt better prepared after the session.

## Description of the case

The University of Manchester MB ChB programme operates over a wide geographical footprint for student placements across the Northwest of England. 18.3% in the city where the medical school is located do not speak English as their main language, which is twice the national average. 18.4% of the city’s population feel they cannot speak English well, with the top three languages spoken being Urdu, Arabic and Polish (19). In contrast, 98.4% of the population in rural Derbyshire, where students also attend placements, speaks English as their main language. The University of Manchester MB ChB Community Team noted, in student written evaluation data, that some GP Clinical Placement Supervisors (CPS) in ethnically diverse areas were consulting in non-English languages. However, student evaluation also revealed the challenge for them of not understanding the content of the GP-patient conversation, resulting in a negative experience for them. The “Mind your Language” (MYL) Protocol was developed to address this issue. The MYL Protocol is a guidance document developed by the Community Team in 2010, outlining to GP CPS how to maximize learning opportunities when students sit in non-English consultations. Here, we explore the development and application of the MYL intervention in creating a tailored CPS training workshop, aimed at empowering them to enhance the experience of medical students.

## Materials and methods

### Intervention design: mind your language training and educational package (protocol, training, resources and experiential learning)

The Community Team considered the easiest way to guide CPS to enhance student experience of non-English consultations. They created a protocol which listed practical steps for the practice and CPS to adhere to, to ensure an excellent student experience (been submitted to UoM Figshare 10.48420/28926224.v1). These were based on recommendations by the lead author, who successfully supervised students in a setting with a high number of non-English consultations. The protocol covered recommendations for before the student attends the practice, key areas to cover in induction, and essential tips before, during and after each consultation. The team followed up the protocol by developing a 75 min training workshop for CPS, covering the issues that students faced and solutions to enhance their experience. The main resource for the training consisted of two pre-recorded simulated videos in a non-English language, using actors. The first video script was based on student evaluation, which described the negative experience they had during the consultation. The second video script had the GP CPS applying tools from the MYL Protocol into the consultation, to enhance the student experience. CPS attending the training session were asked to observe the first video, as if they were students sitting in the consultation, and then to reflect and discuss their feelings of the experience. They then viewed the second video, which applied recommendations from the MYL Protocol, and reflected on whether their feelings improved observing this consultation. These videos with actors were conducted in a made up language. They were later deleted in 2022 as they were not felt to be authentic and replaced by new videos created with a real GP, real patient and a real language.

### CPS recruitment to MYL training session

The MYL Training Session was delivered at the annual GP CPS Away Day in 2010. The Away Day was an opportunity for GP CPS to find out about changes in the MB ChB Programme, educator opportunities, continued professional development (CPD) and networking. The MYL Training Session formed part of a collection of workshops that GP CPS could pre-book for. The team wrote a workshop outline, explicitly stating that this was particularly for GP CPS to attend who consulted in non-English languages. Ten GP CPSs attended the session, and all were from practices where consultations could be in non-English languages.

### Evaluation of intervention

GP CPSs who had attended the MYL Training Session, had good student evaluation scores (overall score 4.5/5 or above) and were located close to the medical school, were emailed an invitation to participate in an intervention evaluation study. We selected high scoring practices as we wanted to assess the intervention in a good quality learning environment. Three practices were recruited, and they were sent the MYL Protocol and a written briefing document outlining the intervention evaluation study and CPS/practice requirements before students attended. The briefing document provided clear instructions for the CPS to outline demographics of the practice population, including ethnicity, language, and any cultural issues during induction. It also advised that the CPS apply the MYL guidance to patient consultations. A key task was for each GP CPS to enable each student to speak to a patient themselves, with the GP CPS as the interpreter.

The MB ChB Programme in Year 3 in 2012 included 6 days in the Nutrition, Metabolism and Endocrine Module for students to attend structured teaching, as well as non-GP placements in the community. The non-GP placements were selected by students, who had the choice of a wide variety of placements. The authors included the MYL hybrid pilot in the menu of opportunities for students to select. Six Year 3 MB ChB students signed up to the pilot. They attended three local GP surgeries in the morning in pairs, where they observed GP consultations in non-English languages. They were provided with a worksheet, to enable them to reflect on their experience while on placement. The content of the worksheet can be found in the CPS Document, that was provided to CPS (10.48420/29023964 UoM Figshare- submitted here and awaiting confirmation).

Students then attended a group debrief session in the afternoon in the medical school. This session consisted of an opportunity for the students to verbally reflect on their earlier clinical experience in a protected small group setting. The session was facilitated by an experienced academic within the Community Team. This provided an opportunity to interrogate the experience and explore how effectively the MYL Protocol was implemented by the GP CPSs. Students were provided with printed copies of the MYL Protocol during the session, to help them with this exercise. The teaching also included the facilitator using PowerPoint slides to enable students to understand key concepts in equity, diversity and inclusion (EDI), exploring topics such as stereotyping and making assumptions. Written student evaluation of the intervention, completed after the teaching ended, stated that it helped to break down barriers when attending practices where patients consulted in non-English languages and positively improved their perceptions of attending such practices (add to UoM Figshare 10.48420/29024417-awaiting review). Table 1 shows the student evaluation of the GP placement, and Table 2 shows evaluation of the group teaching session. A Likert scale is used to score each intervention.

**TABLE 1:** Please rate the quality of the clinical placement- average 4.2/5 (5 out of 6 responses).

Likert score	Number of responses
Poor 1	0
Some concerns 2	0
Satisfactory 3	1
Good 4	2
Excellent 5	2

**TABLE 2:** Please rate the quality of the debrief teaching session- average 4.67/5 (6 responses out of 6).

Likert score	Number of responses
Poor 1	0
Some concerns 2	0
Satisfactory 3	1
Good 4	0
Excellent 5	5

### Expansion of the intervention

The MYL training package was originally developed as a training tool for GP CPSs. However, the authors realized that student experience in these consultations could only improve if they were empowered to challenge any barriers. They integrated the MYL protocol within online learning materials in the first week of the four-week Year 4 GP Block, with resources covering cultural humility and non –English speaking consultations. Students engaged with online learning while on their GP placement, in preparation for themed case discussion teaching at the end of the week.

The Community Team developed two recorded simulated consultations in Sylheti, a dialect of Bengali, in 2022. This was a new set of videos that were different to the original training videos. They involved a real GP and patient, as opposed to actors. An authentic language was also used, rather than a made-up language. The videos were created by the university filming team and consisted of two versions of the same consultation in a GP surgery, seen from the perspective of a medical student observing the interaction. The first consisted of a consultation without the GP applying the MYL protocol, and the second had the GP incorporating methods shared in the protocol. Students observed the first video and reflected on how they felt in this situation. They then viewed the second video and reflected on whether they felt better in this adapted consultation. There followed discussions about how students could ensure they could maximize their learning in these situations. They also had the opportunity to reflect on individual experiences of observing consultations in non-English languages.

It was essential to ensure that GP CPS responsible for delivering community placements were adequately trained in the practical implementation of the MYL protocol and were aware of its associated benefits. This was achieved through ongoing supervisor training delivered both in-person and through written materials on the online learning platform. Additionally, the MYL protocol was explored during triannual CPS development reviews in those practices with non-English consultations. This provided an opportunity for an academic with oversight of the practice, to review undergraduate supervision at both an individual and practice level. Annual CPS training days also incorporated the MYL protocol, to ensure that CPS and other staff were aware of its application in the clinical setting.

### Positive takeaways of MYL

The MYL protocol was presented at the Society for Academic Primary Care (SAPC) North Conference in 2012, which generated interest from other medical school community teams. Given its innovative nature, the team were invited by colleagues in four other UK medical schools, to deliver the training to their GP CPS. These medical schools subsequently developed bespoke guidance for their CPS, with support of the Community Team.

## Discussion

### Future perspectives

The next step is for wider dissemination and uptake of the MYL protocol, contacting more practices with consultations in non-English languages, and inviting them to a bespoke training session. This would cover the MYL protocol and explore methods by which CPS could maximize the learning potential in the clinical setting, as well as gain feedback on their prior experiences. There needs to be robust evaluation of the impact of the GP CPS training sessions, through qualitative focus group analysis, as well as quantitative analysis of written evaluation.

The session should also include training on key equity, diversity, and inclusion (EDI) elements. such as applying cultural humility during supervision. This is advocated by the Medical Schools Council (MSC) in their guidance document, stating “*Curricula should also identify the impact that prejudice, bias, stigma and microaggressions have on students, staff and patients in healthcare environments and the impact this can have on the delivery of care*” (20).

The authors propose that the expanded version of the MYL training that includes students enables them to effectively consult with patients that only speak non-English languages. Bansal et al. (21) evaluated a consultation skills session using professional interpreters both as interpreters and simulated patients. This session improved student confidence in interpreter consultations and even benefited the GP tutor for future clinical practice. However, the authors suggested bilingual actors to play the simulated patient, as the interpreters found this challenging. A similar model could be incorporated into the themed case discussion teaching, utilizing bilingual actors only, and evaluating the impact through written evaluation. The MYL protocol, when used appropriately, bridges language barriers, fostering equitable patient-centered care whilst also including medical students into the consultation.

### Conceptual or methodological constraints

The main constraints to wider expansion of the work include the reliance on engagement from GP CPS consulting in non-English languages. This would involve ensuring that they attend relevant MYL training and are open to applying the learning in the clinical context. Academics with oversight of relevant practices would also need to actively support the practices, to ensure they adapt their placements to enhance the clinical experience.

Another constraint is ensuring EDI training, particularly consulting with interpreters, is embedded into the medical programme. All medical programmes are constricted spaces, with different elements vying for space in the timetable. There would need to either be incorporation of the training into established communication skills teaching sessions, or negotiation to accommodate new teaching, at the expense of other structured teaching time. Clinical years could offer greater flexibility, with the teaching replacing clinical experience time.

## Data Availability

The original contributions presented in this study are included in this article/supplementary material. The data is also available at repository Figshare at the University of Manchester (Mind your Language Protocol https://doi.org/10.48420/28926224.v1; student feedback https://doi.org/10.48420/29024417.v1; CPS document https://doi.org/10.48420/29023964.v1). Further inquiries can be directed to the corresponding author.
